# A First Insight into the Genetic Diversity and Drug Susceptibility Pattern of* Mycobacterium tuberculosis *Complex in Zhejiang, China

**DOI:** 10.1155/2016/8937539

**Published:** 2016-11-22

**Authors:** Zhengwei Liu, Yu Pang, Songhua Chen, Beibei Wu, Haibo He, Aizhen Pan, Xiaomeng Wang

**Affiliations:** ^1^The Institute of TB Control, Zhejiang Provincial Center for Disease Control and Prevention, Zhejiang, China; ^2^National Center for TB Control and Prevention, Chinese Center for Disease Control and Prevention, Beijing, China

## Abstract

In this study, our aim was to determine the predominant genotypes among the* Mycobacterium tuberculosis* (MTB) strains circulating in Zhejiang Province. In addition, we also sought to determine the potential associations between MTB genotypes and susceptibility to first-line drugs. Out of these isolates, 673 (71.6%) were classified into the Beijing genotype, while the other 267 (28.4%) were from non-Beijing families. The highest proportion of Beijing genotype was found in Huzhou (80.0%) and the lowest in Lishui (48.3%). Statistical analysis revealed that there was a significant difference in the prevalence of Beijing genotype among different regions (*χ* ^2^ = 17.57,* P* = 0.04). In addition, the overall proportions of drug resistance to INH, RIF, SM, and EMB were 13.2% (124/940), 21.8% (75/940), 3.4% (32/940), and 5.9% (55/940) in Zhejiang, respectively. Further comparison revealed that there was no significant difference in drug susceptibility profiles between Beijing and non-Beijing strains (*P* > 0.05). In conclusion, we describe the genetic diversity and drug susceptibility pattern of MTB in Zhejiang for the first time. Our data demonstrate that Beijing genotype is the predominant lineage in Zhejiang, while the distribution of Beijing-genotype strains shows geographic diversity. In addition, no correlation is observed between Beijing genotype and anti-TB drug resistance.

## 1. Introduction

Tuberculosis (TB) caused by* Mycobacterium tuberculosis* complex (MTBC) remains a major national health concern in China [1, 2]. According to the estimation by World Health Organization (WHO), there were approximately 0.83 million incident TB cases annually in China [3]. Because China is a vast country of marked cultural, economic, geographic, and infrastructural contrasts, the prevalence of TB varies geographically [1]. Findings from a recent national survey in China demonstrated that the prevalence of bacterially positive TB in western China was significantly higher than that in eastern China in 2010 (212 per 100 000 population versus 66 per 100 000 population) [1]. This spatial heterogeneity of TB burden in China is associated with the various effectiveness of TB control measures in each province, which may have some impact on the TB epidemiology among different regions [1].

Zhejiang Province is located in southeastern China, covering an area of 101,800 square kilometers and including a population of 55.4 million in 2015. Currently Zhejiang had a reported active pulmonary TB incidence of 68.86/100 000 persons in 2010, which was lower than the national average of 78/100 000 persons [1, 4]. Despite the relative low TB incidence, a huge population of over 50 million people contributed to 30 000 new TB cases reported annually in this province [4]. More importantly, Zhejiang is one of the provinces with the highest population density in China, and there are 545 persons per square km, which was 3.8 times of national average level [5]. Hence, it is meaningful to investigate the molecular epidemiology of TB in such setting with low TB incidence and high population density. Unfortunately, to our best knowledge, no systematic study on genetic diversity and drug susceptibility pattern of MTBC has been performed in this region. In this study, we aimed to determine the predominant genotypes among the MTB strains circulating in Zhejiang Province. In addition, we also sought to determine the potential associations with transmission and susceptibility to first-line drugs.

## 2. Materials and Methods

### 2.1. Ethics Statement

This study was approved by the Ethics Committee of Zhejiang Center for Disease Control and Prevention. All the Patients enrolled in this study had signed an informed consent form.

### 2.2. Bacterial Strains

In 2010, Zhejiang Province carried out the anti-TB drug resistance surveillance in randomly selected 30 counties. On the basis of estimation, every site needed to consecutively enroll at least 31 smear-positive TB patients during the surveillance period. One survey administrator interviewed each enrolled patient with the same questionnaire containing the demographic characteristics and treatment history. A total of 940 MTB isolates were isolated from these smear-positive TB patients. Isolates grown on Lowenstein-Jensen (L-J) medium were transferred to the Provincial TB Reference Laboratory for species identification and drug susceptibility testing (DST).

### 2.3. Species Identification and Drug Susceptibility Testing

DST for isoniazid (INH), rifampicin (RFP), ethambutol (EMB), and streptomycin (SM) was performed with the proportional method recommended by the WHO, and the concentrations of drugs in media were following the guidelines from WHO: INH 0.2 *μ*g/mL, RFP 40 *μ*g/mL, EMB 2 *μ*g/mL, and SM 4 *μ*g/mL [24]. When the growth rate was more than 1% compared with the control, a strain was declared as resistant to the drug. The multidrug resistant (MDR) strains were defined as the strains at least resistant to both INH and RIF. In addition, media supplied with paranitrobenzoic (PNB) acid (500 mg/mL) were used to perform species identification. The strains sensitive to PNB were considered as MTB.

### 2.4. Extraction of Genomic DNA

The freshly cultured bacteria were harvested from the surfaces of L-J media. Then the bacteria cells were resuspended in 500 *μ*L Tris-EDTA (TE) buffer (pH 8.0), followed by heating in 95°C water bath for 1 hour. After centrifugation of cellular debris, the DNA in the supernatant was used for PCR amplification reactions [25].

### 2.5. Genotyping

We performed the spoligotyping analysis with a commercially available kit (Isogen Bioscience BV, Maarssen, Netherlands) [24]. Briefly, the fragment containing DR region was amplified with primers DRa (5′-CCGAGAGGGGACGGAAAC-3′) and DRb (5′-GGTTTTGGGTCTGACGAC-3′). After hybridization with the membrane, the final image was detected with a chemiluminescence system, including the ECL detection liquid (Amersham, Buckinghamshire, United Kingdom) and ECL-Hyperfilm (Kodak, Rochester, NY). The original binary data were submitted to the SITVITWEB database to obtain the spoligotyping pattern [26]. In addition, patterns not identified in SITVITWEB database were further assigned to families and subfamilies by Spotclust (http://tbinsight.cs.rpi.edu/run_spotclust.html).

### 2.6. Data Analysis

Associations among multiple categorical variables were evaluated by the Chi-square test, and the statistical results were expressed as odds ratios (ORs) with 95% confidence intervals (CIs). The forward stepwise logistic regression procedures were used to analyze whether statistically significant covariates identified by univariate analysis were independently associated with Beijing genotype. All calculations were performed in SPSS 11.5 (SPSS Inc., USA). Differences were defined as statistically significant if a *P* value was less than 0.05.

## 3. Results

### 3.1. Distribution of Different Genotypes in Zhejiang

**Figure 1:**
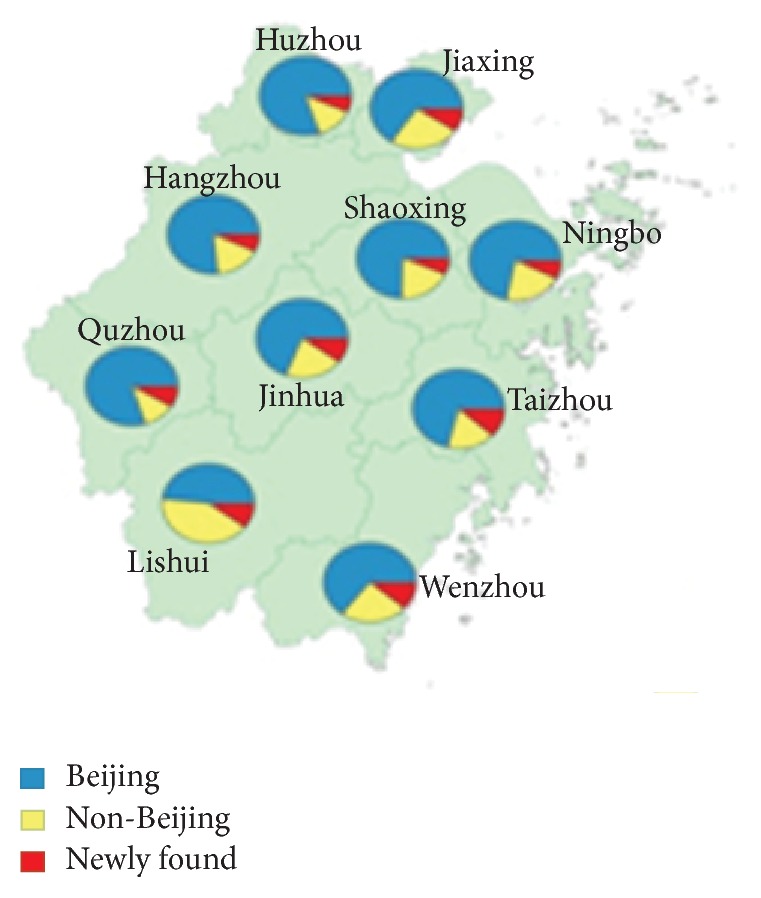
Distribution of Beijing genotype strains in ten prefectures of Zhejiang Province.

A total of 940 representative* M. tuberculosis* isolates were analyzed by spoligotyping in this study. Out of these isolates, 673 (71.6%) were classified into the Beijing genotype, while the other 267 (28.4%) were from non-Beijing families, indicating that the Beijing family is the predominant genotype in Zhejiang Province. Strains belonging to non-Beijing families included 92 strains from the T1 family (9.8%), 47 from the T2 family (5.0%), 13 from the T3 family (1.4%), 10 from the H3 family (1.1%), 7 from the MANU2 family (0.7%), 5 from the U family (0.5%), 9 from others (1.0%), and 84 from newly found genotypes (8.9%). We further analyzed the distribution of Beijing genotype in different districts of Zhejiang. As shown in Figure 1, the highest proportion of Beijing genotype was found in Huzhou (80.0%) and the lowest in Lishui (48.3%). Statistical analysis revealed that there was a significant difference in the prevalence of Beijing genotype among different regions (*χ* ^2^ = 17.57, *P* = 0.04).

### 3.2. Predominant Spoligotypes in Zhejiang

A total of 129 spoligotypes were identified in this study. Among these spoligotypes, 55 spoligotypes were represented as Shared International Types (SITs) based on SITVITWEB database, while the other 74 were reported for the first time (Table 1 and Table S1 available online at http://dx.doi.org/10.1155/2016/8937539). By the analysis with BioNumerics software, 840 (89.4%) isolates were classified into 29 clusters with 2 or more strains. In addition, 100 (10.6%) isolates did not share the same spoligotype with the others.

Out of the 29 clusters, the most prevalent spoligotype was SIT1 belonging to Beijing family, accounting for 68.3% of all isolates (642/940). After ST1, ST53 (5.6%, 53/940), a member of T1 family, was the second most frequent spoligotype in Zhejiang. The third largest lineage was SIT52, assigned to the T2 family, with 40 strains (4.3%). In addition, the numbers of SIT190 (Beijing family), SIT37 (T3 family), SIT521 (T1 family), and SIT334 (T1 family) were 15 (1.6%), 12 (1.3%), 9 (1.0%), and 8 (0.9%) (Table 1).

### 3.3. Drug Susceptibility Profiles of Beijing Genotype and Non-Beijing Genotype

We further analyzed the difference of drug susceptibility profiles between Beijing and non-Beijing genotype strains. As shown in Table 2, the overall proportions of drug resistance to INH, RIF, SM, and EMB were 13.2% (124/940), 21.8% (75/940), 3.4% (32/940), and 5.9% (55/940) in Zhejiang, respectively. In addition, out of these isolates, 55 (5.9%) isolates were classified to MDR. Further comparison of DST results between Beijing and non-Beijing family showed that there was no significant difference in drug susceptibility profiles between strains in these two families (*P* > 0.05).

### 3.4. Demographic Characteristics of TB Patients Infected by Strains of Beijing and Non-Beijing Family

A classification of patients infected with Beijing genotype strains, stratified according to sex, age, treatment history, residence, and occupation, was shown in Table 3. Overall, the proportion of men infected by Beijing genotype strain was similar to that of women (*P* = 0.97). In addition, age, treatment history, residence, and occupation had no influence on the prevalence of Beijing genotype strains in Zhejiang (*P* > 0.05). In the multivariate analysis by a stepwise multiple regression, no variable was statistically significant, indicating that the infection of Beijing genotype had no correlation with various demographic characteristics and treatment history.

## 4. Discussion

This is the first study to investigate the population structure and the relationship between Beijing genotype and DST profiles in Zhejiang Province. Our data demonstrated that Beijing family strain was still the predominant MTB genotype in Zhejiang Province. Evidences from molecular epidemiological studies have confirmed that MTB Beijing genotype is the most successful clade circulating in China [24], while the distribution of Beijing genotype exhibits geographic diversity in various regions of China [24]. Overall, the proportion of Beijing genotype in northern China is significant higher than that in southern China [24]. Although the prevalence of Beijing genotype among most of northern regions was higher than 75%, we also found that Beijing genotype only accounted for 57.3% of MTB isolates in Xinjiang Uygur Autonomous Region [14]. In southern China, the prevalence of Beijing family in Zhejiang (71.6%) was lower than that in Jiangsu (80.5%) [16], Shanghai (79.3%) [17], and Jiangxi (76.7%) [18], while it was higher than that in Chongqing (66.7%) [20], Guangxi (61.9%) [21], Fujian (57.3%) [22], and Guizhou (54.0%) [6–13, 15, 19, 23] (Table 4).

In addition, lower prevalence of Beijing genotype was observed in Lishui when compared with other regions. On one hand, Lishui is located in the mountain area, which is relatively undeveloped in Zhejiang Province [27]. Due to the unsatisfactory public health setting, the rate of BCG vaccination among children in the 1990s in Lishui was lower than that in other regions of Zhejiang. Numerous literatures reported that BCG vaccination might be a positive selective force favoring the spread of the Beijing genotype [28, 29]. Hence, we hypothesize that the low proportion of BCG vaccination may be responsible for the low prevalence of Beijing genotype in this area. On the other hand, the small sample size in Lishui may be another potential issue resulting in bias. Further analysis is essential to investigate the population structure of MTB in this region with more MTB isolates.

Drug resistance surveillance is considered as an important tool for understanding the prevalence of drug resistance in a country or region and for formulating proper strategies for drug resistant TB control [30]. In this study, our data demonstrated that the prevalence of MDR-TB in Zhejiang was 5.9%, which is lower than the national level (8.3%) [2] and that in Jiangsu (16.6%) [31], whereas it is higher than that in Shanghai (4.0%) [32]. The relatively low proportion of MDR-TB in Zhejiang may be attributed to several reasons. First, low prevalence of MDR in Zhejiang may be due to the successful implementation of directly observed treatment short course (DOTS). DOTS strategy has become the internationally recommended approach for TB control programs [33]. The effective DOTS implementation leads to reducing emergence of drug resistance among TB patients [34]. In 2001, DOTS strategy was integrated into the local TB control programme of Zhejiang, where the coverage rate of DOTS strategy was higher than 95% since 2001. Hence, the successful DOTS implementation may be the major reason associated with the low prevalence of MDR in Zhejiang. Second, application of sensitive diagnostic methods may improve diagnosis and treatment of TB in Zhejiang, resulting in decrease of MDR-TB. Under the local financial support, the capabilities of TB laboratories in Zhejiang were strengthened significantly since 2004. Compared with other provinces, mycobacterial culture and conventional DST were performed as routine work in all county and prefectural TB laboratories of Zhejiang in 2005, respectively. The application of these sensitive diagnostic methods would improve case detection and prompt initiation of appropriate treatment, thereby reducing the emergence of drug resistant TB.

Numerous literatures have reported that Beijing genotype stains have significant associates with drug resistance, which might be responsible for the spread and emergence of MDR-TB [24, 28]. In contrast, a recent literature from Yang et al. revealed that Beijing genotype strains were significantly associated with recent transmission but were not associated with drug resistance [35]. In line with the latter studies, our data showed that there was no significant difference in the proportion of drug resistance between Beijing genotype and non-Beijing genotype. There were several potential reasons responsible for this observation. On one hand, Beijing genotype strains have two major evolutionary lineages, ancient and modern Beijing genotypes [29]. Despite sharing the similar spoligotyping profiles, these two lineages displayed different pathogenic and drug resistant features [29]. Hence, the different proportion of modern and ancient Beijing genotype subpopulations in different settings may serve as an important reason for the discrepancy of the association between Beijing genotype and drug resistance profiles. On the other hand, non-Beijing genotype strains include a variety of sublineages, such as T1, T2, CAS, LAM, and MANU2. Different non-Beijing sublineages exhibit diverse correlation with drug susceptibility, even within the same sublineage. A recent study from Lukoye et al. has revealed that the T2 MTB genotype is associated with anti-TB drug resistance [36], whereas T2 family strains isolated from Uganda has no statistical relation with anti-TB drug resistance [37]. We hypothesize that this difference is due to the poor discriminatory power of spoligotyping. Further molecular epidemiological study with MIRU-VNTR will help us to investigate the relationship between genotype and drug resistance.

We also realized that there were several obvious limitations in our study. First, although spoligotyping provides adequate effectiveness for distinguishing Beijing and non-Beijing genotype strains, its low discriminatory power makes spoligotyping insufficient for epidemiological linking studies [38]. Currently, another PCR-based method named mycobacterial interspersed repetitive units-variable numbers of tandem repeats (MIRU-VNTR) shows favorable discriminatory capacity when compared with IS6110-RFLP profiling [38]. Further genotyping analyses with MIRU-VNTR will extend our knowledge of the transmission profiles of MTB strains circulating in Zhejiang Province. Second, Beijing genotype is associated with fluoroquinolone (FQ) resistance, as evidenced in several studies [39, 40]. Unfortunately, drug susceptibility testing was performed for only four first-line drugs rather than FQs and second-line injectable drugs, which impedes further investigation on the relationship between Beijing genotype and FQ resistance in Zhejiang.

In conclusion, we describe the genetic diversity and drug susceptibility pattern of MTB in Zhejiang for the first time. Our data demonstrate that Beijing genotype is the predominant lineage in Zhejiang, while the distribution of Beijing genotype strains shows geographic diversity. In addition, no correlation is observed between Beijing genotype and anti-TB drug resistance. The relatively low prevalence of MDR-TB in Zhejiang reflects the achievement of successful DOTS implementation, while further application of molecular diagnostic tools in the routine diagnosis algorithm will improve case detection and prompt initiation of appropriate treatment, thereby reducing the emergence of drug resistant TB.

## Supplementary Material

Table S1 Identification of new found spoligotypes by SpotClust.

## Figures and Tables

**Table 1:** Distribution of spoligotypes shared by *Mycobacterium tuberculosis* circulating from Zhejiang Province.

SpolDB4 ID^*∗*^	Spoligotype binary description	SIT^†^	Number (%)
Beijing	□□□□□□□□□□□□□□□□□□□□□□□□□□□□□□□□□□■■■■■■■■■	1	642 (68.3)
	□□□□□□□□□□□□□□□□□□□□□□□□□□□□□□□□□□■■■■■□■■■	190	15 (1.6)
	□□□□□□□□□□□□□□□□□□□□□□□□□□□□□□□□□□□□■■■■■■■	269	2 (0.2)
	□□□□□□□□□□□□□□□□□□□□□□□□□□□□□□□□□□■□■■■■■■■	621	3 (0.3)
	□□□□□□□□□□□□□□□□□□□□□□□□□□□□□□□□□□■■■□■■■■■	632	6 (0.6)
	□□□□□□□□□□□□□□□□□□□□□□□□□□□□□□□□□□■■■■■■□■■	941	1 (0.1)
	□□□□□□□□□□□□□□□□□□□□□□□□□□□□□□□□□□■■■■□□■■■	1168	2 (0.2)
	□□□□□□□□□□□□□□□□□□□□□□□□□□□□□□□□□□■■■□□■■■■	1364	1 (0.1)
	□□□□□□□□□□□□□□□□□□□□□□□□□□□□□□□□□□■■■■■■■□■	1674	1 (0.1)

H3	■■■■■■■■■■■■■■■■■■■■■■■■■■■■■■□■□□□□■■■□■■■	49	1 (0.1)
	■■■■■■■■■■■■■■■■■■■■■■■■■■■■■■□■□□□□■■■■■■■	50	6 (0.6)
	■■■■■■■■■■■■■■■■■■□■■■■■■■■■■■□■□□□□■■■■■■■	183	2 (0.2)
	■■■■■■■■■■■■■■■■■■■■■■■■■■■■■□□■□□□□■■■■■■■	390	1 (0.1)

H4	■■■■■■■■■■■■■■■■■■■■■■■■■■■■□□□■□□□□■■■□■■■	817	1 (0.1)

LAM9	■■■■■■■■■■■■■■■■■■■■□□□□■■■■■■■■□□□□■■■■■■■	42	1 (0.1)

MANU2	■■■■■■■■■■■■■■■■■■■■■■■■■■■■■■■■□□■■■■■■■■■	54	6 (0.6)
	■■■■■■■■■■■■■■■■■■■■■■■■■■■■■■□■□□■■■■■■■■■	1634	1 (0.1)

S	■■■■■■■■□□■■■■■■■■■■■■■■■■■■■■■■□□□□■■■■■■■	34	1 (0.1)

T1	■■■■■■■■■■■■■■■■■■■■■■■■■■■■■■■■□□□□■■■□□□□	51	1 (0.1)
	■■■■■■■■■■■■■■■■■■■■■■■■■■■■■■■■□□□□■■■■■■■	53	53 (5.6)
	■■■■□■■■■■■■■■■■■■■■■■■■■■■■■■■■□□□□■■■■■■■	154	2 (0.2)
	■■■■■■■■■■■■■■■■■■■■■■■■■■■■■□■■□□□□■■■■■■■	167	1 (0.1)
	■■■□■■■■■■■■■■■■■■■■■■■■■■■■■■■■□□□□■■■■■■■	205	1 (0.1)
	■■■■■■■■■■■■■■■■■■■■■■■■■■■■■■■■□□□□■■■■■□■	278	1 (0.1)
	■■□□■■■■■■■■■■■■■■■■■■■■■■■■■■■■□□□□■■■■■■■	285	1 (0.1)
	■□■■■■■■■■■■■■■■■■■■■■■■■■■■■■■■□□□□■■■■■■■	334	8 (0.9)
	■■■■■■■■■■■■■□□□□■□□□□□■■■■■■■■■□□□□■■■■■■■	379	1 (0.1)
	■■■■■■■■■■■■■□■■■■■■■■■■■■■■■■■■□□□□■■■■■■■	393	2 (0.2)
	■■■■■■■■■■■■■■■■■■■■■■■■■■■■■■■■□□□□■□■■■■■	520	2 (0.2)
	■■■■■■■■■■■■■■■■■■■■■■■■■■■■■■■■□□□□■■□□□■■	521	9 (1.0)
	■■■■■■■■■■■■■■■■■■■■■■■■■■■■■■■■□□□□■■■■□■■	612	1 (0.1)
	■■■■■■■■■■■■■□□□□□□□□□□□■■■■■■■■□□□□■■■■■■■	803	1 (0.1)
	■□□■■■■■■■■■■■■■■■■■■■■■■■■■■■■■□□□□■■■■■■■	804	1 (0.1)
	■■■■□■■■■■■■■■■■■■■■■■■■■■■■■■■■□□□□■■■□□□□	833	1 (0.1)
	■■■■■■■■■■■■■■■■■■■■■■■■■■■■■■■■□□□□■■□□■■■	888	2 (0.2)
	■■■■■■■■■■■■■□□□■■■■■■■■■■■■■■■■□□□□■■■■■■■	913	1 (0.1)
	■■■■■■■■■■□■■■■■■■■■■■■■■■■■■■■■□□□□■■■■■■■	917	1 (0.1)
	■■■■■■■■■■■■■■■■■■■□□□□□□■■■■■■■□□□□■■■■■■■	1688	1 (0.1)
	■■■■■■■■■■■■■■■■■■■■■■■■■■□■■■■■□□□□■■■■■■■	1626	1 (0.1)

T2	■■■■■■■■■■■■■■■■■■■■■■■■■■■■■■■■□□□□■■■□■■■	52	40 (4.3)
	■■■■■■■■■■■■■■□■■■■■■■■■■■■■■■■■□□□□■■■■■■■	118	1 (0.1)
	■■■■■■■■■■■■■■■■■■■■■■■■■■□■■■■■□□□□■■■□■■■	515	1 (0.1)
	■■■■■■■■□■■■■■■■■■■■■■■■■■■■■■■■□□□□■■■□■■■	1265	1 (0.1)
	■□■■■■■■■■■■■■■■■■■■■■■■■■■■■■■■□□□□■■■□■■■	1302	2 (0.2)
	■■□■■■■■■■■■■■■■■■■■■■■■■■■■■■■■□□□□■■■□■■■	1332	1 (0.1)
	□□■■■■■■■■■■■■■■■■■■■■■■■■■■■■■■□□□□■■■□■■■	1613	1 (0.1)

T2-T3	■■■■■■■■■■■■□■■■■■■■■■■■■■■■■■■■□□□□■■■□■■■	73	3 (0.3)

T3	■■■■■■■■■■■■□■■■■■■■■■■■■■■■■■■■□□□□■■■■■■■	37	12 (1.3)
	■■■■■■■■■■■■□■□■■■■■■■■■■■■■■■■■□□□□■■■■■■■	1547	1 (0.1)

T4	■■■■■■■■■■■■■■■■■■□■■■■■■■■■■■■■□□□□■■■■■■■	40	1 (0.1)

U	■■■■■■■■■■■■■■■■■■■■■■■□□□□□□□□□□□□□□□■■■■■	232	1 (0.1)
	■■■■■■■■■■■■■■■■■■■■■■■■■■■■■■■■■■■■■■■□■■■	246	1 (0.1)
	■■■■■■■■■■■■■■■■■■■■■■■■■■■■■■■■□□□□□■■□■■■	1098	1 (0.1)
	□□□□□□□□□□□□□□□□□□□□□□□□□□□□□□□□□□■■■■■□□□□	1311	2 (0.2)

U (like H)	■■■■■■■■■■■■■■■■■■■■■■■■□□□□□□□□□□□□□□□□□□□	46	2 (0.2)

Newly found	—		84 (8.9)

^*∗*^Representing spoligotype families annotated in SITVITWEB database.

^†^SIT from SITVITWEB database.

**Table 2:** Difference of drug susceptibility between Beijing and non-Beijing families.

Characteristic	Number of isolates (%)			*P* value	OR	95% CI
	Total (*n* = 940)	Beijing (*n* = 673)	Non-Beijing (*n* = 267)			
Resistance^a^						
INH	124 (13.2)	86 (12.8)	38 (14.2)	0.55	0.88	0.59–1.33
RIF	75 (8.0)	53 (7.9)	22 (8.2)	0.85	0.95	0.57–1.60
SM	205 (21.8)	140 (20.8)	65 (24.3)	0.24	0.82	0.58–1.14
EMB	32 (3.4)	24 (3.6)	8 (3.0)	0.66	1.20	0.53–2.70
MDR	55 (5.9)	40 (5.9)	15 (5.6)	0.85	1.06	0.58–1.96
Four-drug susceptibility	668 (71.1)	489 (72.7)	179 (67.0)	0.09	1.31	0.96–1.78
Four-drug resistance	18 (1.9)	13 (1.9)	5 (1.9)	0.95	1.03	0.36–2.92

^a^INH: isoniazid; RIF: rifampicin; SM: streptomycin; EMB: ethambutol; MDR: multidrug resistance.

^b^OR: odds ratio; 95% CI: 95% confidence interval.

**Table 3:** Difference of demographic characteristics between Beijing and non-Beijing families.

Characteristics	Number of isolates (%)		*P* value	OR	95% CI
	Beijing	Non-Beijing			
Sex					
Men	210 (31.2)	83 (31.1)	Reference	—	—
Women	463 (68.8)	184 (68.9)	0.97	1.00	0.73–1.35
Age					
0–24	119 (17.7)	38 (14.2)	Reference	—	—
25–34	136 (20.2)	56 (21.0)	0.30	0.78	0.48–1.25
35–44	116 (17.2)	57 (21.3)	0.08	0.65	0.40–1.50
45–54	101 (15.0)	39 (14.6)	0.47	0.83	0.49–1.39
55–64	68 (10.1)	32 (12.0)	0.17	0.68	0.39–1.18
≥65	133 (19.8)	45 (16.9)	0.82	0.94	0.57–1.55
Treatment history					
New case	607 (90.2)	237 (88.8)	Reference	—	—
Retreated	66 (9.8)	30 (11.2)	0.51	0.86	0.54–1.36
Population					
Permanent	253 (37.6)	105 (39.3)	Reference	—	—
Migrant	420 (62.4)	162 (60.7)	0.62	1.08	0.80–1.44
Occupation					
Farmer	318 (47.3)	123 (46.1)	Reference	—	—
Worker	215 (31.9)	90 (33.7)	0.63	0.92	0.67–1.28
Others	140 (20.8)	54 (20.2)	0.99	1.00	0.69–1.46

**Table 4:** Prevalence of Beijing genotype strains in different regions of China.

Region	Number of isolates		Prevalence (%)	Reference
	Total	Beijing		
⁡Northern China				
Gansu	445	409	91.9	[6]
Hebei	422	384	91.0	[7]
Henan	197	177	89.8	[8]
Heilongjiang	200	179	89.5	[9]
Shaanxi	195	165	84.6	[10]
Beijing	1585	1300	82.0	[11]
Inner Mongolia	372	294	79.0	[12]
Shandong	206	160	77.7	[13]
Xinjiang	379	217	57.3	[14]
Southern China				
Tibet	576	522	90.6	[15]
Jiangsu	246	198	80.5	[16]
Shanghai	396	314	79.3	[17]
Jiangxi	133	102	76.7	[18]
Zhejiang	940	673	71.6	This study
Sichuan	306	212	69.3	[19]
Chongqing	297	198	66.7	[20]
Guangxi	176	109	61.9	[21]
Fujian	234	134	57.3	[22]
Guizhou	265	143	54.0	[23]
China	4017	2500	62.2	[24]
