# Supplementary material for: A First Insight into the Genetic Diversity and Drug Susceptibility Pattern of Mycobacterium tuberculosis Complex in Zhejiang, China
Source: Biomed Res Int. 2016 Nov 22;2016:8937539. doi: 10.1155/2016/8937539 (PMC5138472; doi:10.1155/2016/8937539)
Supplement: Supplementary file 1 — Table S1 Identification of new found spoligotypes by SpotClust. [file 8937539.f1.docx]

**Supplemental Table 1 Identification of new found spoligotypes by SpotClust**

| Spoligotype description binary | Family | No. of isolates |
| --- | --- | --- |
| □□□□□□□□□□□□□□□□□□□□□□□□□□□□□□□□□□■■■■□□□□□ | Beijing | 1 |
| □□□□□□□□□□□□□□□□□□□□□□□□□□□□□□□□□□■■□□□■■■■ | Beijing | 1 |
| □□□□□□□□□□□□□□□□□□□□□□□□□□□□□□□□□□■■■■■■□□□ | Beijing | 1 |
| □□□□□□□□□□□□□□□□□□□□□□□□□□□□□□□□□□■■■□□□□■■ | Beijing | 1 |
| ■■■■■■■■■■■■■■■■■■■■■□□□□■■■■■■■□□■■■■■■■■■ | Family33 | 1 |
| ■■■■■■■■■■■■■□□□■■■■■■■■■■■■■■■■□□■■■■■■■■■ | Family33 | 1 |
| ■■■■■■■■■■■■■■■■■■■■■□■■■■■■■■■■□□■■■■■■■■■ | Family33 | 1 |
| ■■■■■■■■■□□□□■□□□□□□□□□□■■■■■■■■■■■■■■■□■■■ | Family33 | 1 |
| ■■■■□■■■■■■■■■■■■■■■■■■■■■■■■■□■□□■■■■■■■■■ | Family33 | 1 |
| ■■■■■■■■■■■■□■■■■■■□□■■■□□□□□□□□□□□□□□□□□□□ | Family34 | 1 |
| ■■■■■■■■■■■■■■■■■■□□□□□□□□□□□□□□□□□□□□□□□□□ | Family34 | 1 |
| □□□□□□□□□□□□□□□□□□□□□□□□□□□□□□■■□□□□■■■■■■■ | Family36 | 1 |
| ■■■■■■■■■■■■□□□□□■□□□■■■■■■■■■■■□□□□■■■■■■■ | H37Rv | 1 |
| ■■■■■■■■■■■□■□□□□■□□□■■■■■■■■■■■□□□□■■■■■■■ | H37Rv | 1 |
| ■■■□■■■■■■■■■■■■■■■■■■■■■□□□□□■■□□□□■■■■■■■ | Haarlem1 | 2 |
| □□□□■■■■■■■■■■■■■■■■■■■■□□□□□□□■□□□□■■■■■■■ | Haarlem1 | 1 |
| ■■□■■■■■■■■■■■■■■■■■■■■■□□□□□□□■□□□□■■■■■■■ | Haarlem1 | 1 |
| ■■■■■■■■■■■■□■■■■■■■■■■■□□□□□□□■□□□□■■■■■■■ | Haarlem1 | 1 |
| ■■■■■■■■■■■■■■□■■■■■■■■■□□□□□□□■□□□□■■■■■■■ | Haarlem1 | 1 |
| □■■■■■■■■■■■■■■■■■■■■■■■□□□□□□□■□□□□■■■□■■■ | Haarlem1 | 4 |
| ■■■■■■■■■■■■■■■■■■■■■■■■□□□□□□□□□□□□■■■■□□□ | Haarlem1 | 1 |
| ■■■■■■■■■■■■■■■■■■■■■□□□□■■■■■■■□□□□■■■□■■■ | LAM10 | 1 |
| ■■■■□■■■■■■■■■■■■■■■■□□□□■■■■■■■□□□□■■■■■■■ | LAM10 | 1 |
| ■■■■■■■■■■■■■■■■■■■■■□□□□■■■■■■■□□□□■■■■■■■ | LAM10 | 1 |
| ■■■□□■■■■■■■■■■■■■■■■■□□□■■■■■■■□□□□■■■□■■■ | LAM10 | 1 |
| ■■■■■■■■■■■■■■■■□■□□□□□□□□□□■■■■□□□□■■■□■■■ | LAM9 | 1 |
| ■■■■■■■■■■■■■□□□□■□□□□□□■■■■□□□■□□□□■■■■■■■ | LAM9 | 1 |
| ■■■■■■■■■■■■■□□□□■□□□□□□■■■■■■■■□□□□■■■■■■■ | LAM9 | 1 |
| □□■■■■■■■■■■■■■■■■■■□□□□□■■■■■■■□□□□■■■□□■■ | LAM9 | 1 |
| ■■■■■■■■■■■■■■■■■■■■□□□□□■■■■■■■□□□□■■■□□■■ | LAM9 | 3 |
| ■■■■■■■■■■■■■■■■■■□□□□□□■□□□■■■■□□□□■■■■■■■ | LAM9 | 1 |
| ■■■■■■■■■■■■■□□□□■□□□□□□■■■■■■■□□□□□■■■■■■■ | LAM9 | 1 |
| ■■■■■■■■□□□■■■■■■■■■■■■■■■■■■■■■□□□□■■■□■■■ | S | 1 |
| ■■■■■■■■■■■■□■■■■■■■■□□□■■■■■■■■□□□□■■■□■■■ | T1 | 1 |
| ■■■□□■■■■■■■■■■■■■■■■■■□□□■■■■■■□□□□■■■■■■■ | T1 | 1 |
| ■■■■■■■■■■■■■□□□■■■■■■■■■■■■□■■■□□□□■■■■■■■ | T1 | 1 |
| ■■■□□□□□□■■■■■■■■■■■■■■■■■■■■■■■□□□□■■■■■■■ | T1 | 1 |
| ■■■■■■■■■■■■■■■■□■■■■■■■■■■■■■■■□□□□□□■■■■■ | T1 | 1 |
| ■■■■■■■■■■■□□□□■■■■■■■■■■■■■■■■■□□□□■■■□■■■ | T1 | 2 |
| ■■■■■■■■■■■■■■■■■■■■■■■□■□□□□■□□□□□□□□□■■■■ | T1 | 1 |
| ■■■■■■■■■■■■■■■■■■■■■■■□□□□□□■■■□□□□■■■□■■■ | T1 | 1 |
| ■■■■■■■■■■■■■■■■□■■■■■■■■■■■■■■□□□□□■■■□□■■ | T1 | 1 |
| ■■■■■■■■■■■■■■■■■■■■■■■■■■■□□□■■□□□□■■■■■■■ | T1 | 1 |
| ■■■■■■■■■■■■■■■■■■■■■■■■■■□■■□■■□□□□■■■■■■■ | T1 | 1 |
| ■■■□■■■■■■■■■■■■■■■■■■■■■■■■■■■□□□□□□□□■■■■ | T1 | 1 |
| ■■■■■■■■■■■■□■■■■■■■■■■■■■■■■■■□□□□□■■■■■■■ | T1 | 1 |
| ■■■□■■■■■■■■■■■■■■■■■■■■■■■■■■■□□□□□■■■■■■■ | T1 | 1 |
| ■■■■■■■■■■■■■■■■■■■■■■■■■■■■■□■■□□□□□□□■■■■ | T1 | 1 |
| ■□□■■□■■■■■■□■■■■■■■■■■■■□□■■■■■□□□□■■■□■■■ | T1 | 1 |
| ■□□□□□□■■■■■□■■■■■■■■■■■■■■■■■■■□□□□■■■■■■■ | T1 | 1 |
| ■■■□□■■■■■■■■■■■■■■■■■■■□□□□■■■■□□□□■■■□■■■ | T1 | 1 |
| ■■■■■■■■■■■■■■■■■■■■■■□■□□■■■■■■□□□□■■■■■■■ | T1 | 1 |
| ■□■■□■■■■■■■□□□■■■■■■■■■■■■■■■■■□□□□■■■■■■■ | T1 | 1 |
| ■■□■■■■■■■■□□□■■■■■■■■■■■■■■■■■■□□□□■■■■■■■ | T1 | 1 |
| □□□□□■■■■■■■□■■■■■■■■■■■■■■■■■■■□□□□■■■□■■■ | T1 | 1 |
| □□□■■■■■■■■■□■■■■■■■■■■■■■■■■■■■□□□□■■■□■■■ | T1 | 2 |
| ■■■■■■■■■■■■■■■■■■□■■■□■■■■■■■■■□□□□■■■■■■■ | T1 | 1 |
| ■■■■■■■■■■■□■■■■■■■■■■□■■■■■■■■■□□□□■■■■■■■ | T1 | 1 |
| ■■■■■■■■■■□■■■■■■■■■■■■■■■■■■■■■□□□□■■□■■■■ | T1 | 2 |
| □■■■■■□■■■■■■■■■■■■■■■■■■■■■■■■■□□□□■■■■■■■ | T1 | 1 |
| ■■□□□□■■■■■■■■■■■■□■■■■■■■■■■■■■□□□□■■■□■■■ | T1 | 1 |
| ■■■■■□□□□■□■■■■■■■■■■■■■■■■■■■■■□□□□■■■□■■■ | T1 | 1 |
| ■■■■■■■■■■■■■■■■■■■■■□■■■■■■■■■■□□□□□□■□■■■ | T1 | 1 |
| ■■■■■■■■■■■■■■■■■■■■■■□■■■■■■■■■□□□□□□■□■■■ | T1 | 1 |
| ■■■■■■■■■■■■■■■■■■■■■■■■■■■■■■■■□□□□□□■□■■■ | T1 | 1 |
| ■■■■■■■□■■■■■■■■■■□■■■■■■■■■■■■■□□□□■■■□■■■ | T1 | 2 |
| ■■■■■■■■■■■■■■□□■■■■■■■■■■■■■■■■□□□□■■■□■■■ | T1 | 1 |
| ■□■■■■■■■■■■■■■■■■■■■■■■■■■■■■■■□□□□■■■□□□□ | T2 | 1 |
| ■■■■■■■■■■■■□■■■■■■■■□□■■□□□■■■■□□□□■■■□□□□ | T2 | 1 |
| ■■■■■■■■□□□□□□□□■■■■■■■■■■■■■■■■□□□□■■■■■■■ | T3 | 1 |
| ■■■■■■■□□□□□□□□□□□□■■□□□□□□□□□□■□□□□■■■■■■■ | T3 | 1 |
| ■□□□□□□□□□□□□□□□□□□□□■■■■■■■■■■■□□□□■■■■■■■ | T3y | 1 |
| ■■■■■■□■■■■■■□□□□□□□□□□□■■■■■■■■□□□□■■■■■■■ | T4 | 1 |
| ■□□□□□□□□□□■■■■■■■■■■■■■■■■■■□■■□□□□■■■■■■■ | X3 | 1 |
